# Algal Bloom Prediction Using Extreme Learning Machine Models at Artificial Weirs in the Nakdong River, Korea

**DOI:** 10.3390/ijerph15102078

**Published:** 2018-09-21

**Authors:** Hye-Suk Yi, Sangyoung Park, Kwang-Guk An, Keun-Chang Kwak

**Affiliations:** 1 Department of Bioscience and Biotechnology, Chungnam National University, Daejeon 34134, Korea; yihs@kwater.or.kr; 2 K-Water Convergence Institute, Korea Water Resources Corporation, Daejeon 34350, Korea; sypark@kwater.or.kr; 3 Department of Control and Instrumentation Engineering, Chosun University, Gwangju 61452, Korea

**Keywords:** Nakdong River, regulated river, harmful algal bloom, environmental management, prediction modeling, extreme learning machine, ANFIS

## Abstract

In this study, we design an intelligent model to predict chlorophyll-a concentration, which is the primary indicator of algal blooms, using extreme learning machine (ELM) models. Modeling algal blooms is important for environmental management and ecological risk assessment. For this purpose, the performance of the designed models was evaluated for four artificial weirs in the Nakdong River, Korea. The Nakdong River has harmful annual algal blooms that can affect health due to exposure to toxins. In contrast to conventional neural network (NN) that use backpropagation (BP) learning methods, ELMs are fast learning, feedforward neural networks that use least square estimates (LSE) for regression. The weights connecting the input layer to the hidden nodes are randomly assigned and are never updated. The dataset used in this study includes air temperature, rainfall, solar radiation, total nitrogen, total phosphorus, N/P ratio, and chlorophyll-a concentration, which were collected on a weekly basis from January 2013 to December 2016. Here, upstream chlorophyll-a concentration data was used in our ELM2 model to improve algal bloom prediction performance. In contrast, the ELM1 model only uses downstream chlorophyll-a concentration data. The experimental results revealed that the ELM2 model showed better performance in comparison to the ELM1 model. Furthermore, the ELM2 model showed good prediction and generalization performance compared to multiple linear regression (LR), conventional neural network with backpropagation (NN-BP), and adaptive neuro-fuzzy inference system (ANFIS).

## 1. Introduction

Recent climate change and economic development around the world has been at the cost of environmental deterioration over the same time period, and development has impacted human life directly and indirectly. Among environmental degradation issues, algal blooms refer to the explosive increase and high concentration of phytoplankton in the ecosystem. This has become a challenge facing human society today as algal blooms are becoming more prevalent throughout the world [1,2]. Water quality, hydrology, and climate are the main factors influencing algal blooms in terms of chlorophyll dynamics. Although the general relationship between environmental conditions and phytoplankton dynamics has been extensively studied in the past [3,4,5], controlling algal blooms is difficult because the relationship between environmental factors and algal blooms has different characteristics that depend on geography and time.

In terms of model development, there are typically two types of forecasting models: deductive and inductive models. Deductive models are based on existing theories and knowledge, which enable users to simulate the behavior of various systems. Numerical modeling as a form of deductive modeling has an advantage in forecasting, considering geographical and hydrodynamic variations [6]. However, this method requires numerous input data, calibration, and validation, resulting in uncertainty in the model parameters [7,8]. As an alternative, computational artificial intelligence techniques have been developed as more efficient tools in recent years for predicting or forecasting algal blooms. With the development of artificial intelligence models, an artificial neural network (ANN) was applied to predict algal blooms by assessing eutrophication and simulating chlorophyll-a concentration. Support vector machine and deep learning techniques were also applied to predict phytoplankton abundance. Zhang [2] presented a novel prediction approach for algal blooms based on deep learning to represent and predict highly dynamic and complex phenomena. Tian [3] performed an optimization of a traditional ANN model for predicting chlorophyll dynamics with the goal of decrease the cost of in-situ aquatic environmental monitoring and increase the accuracy of bloom forecasting. Loi [9] attempted to develop an ELM-based predictive model to simulate dynamic changes in phytoplankton abundance in the Macau Reservoir, given a variety of water variables. Rogers [10] presented a new approach to nonlinear groundwater management with the aid of ANNs and optimized aquifer remediation.

Considering the drawbacks of ANNs, extreme learning machine (ELM) was recently thought to be a better solution. Extreme means that its learning speed is extremely fast while it has higher generalization than gradient-descent-based learning [11]. In fact, it has been shown that ELM can be 10 times faster compared to some traditional algorithms with backpropagation [12]. ELM has been used to assess the stability of electric power systems, optimize the lifetime of transportation systems, and predict electrical power. Xu [13] developed an ELM-based predictor for real-time frequency stability assessment to enhance the dynamic security of power systems. Sun [14] proposed a two-stage approach to optimize the lifetime of transportation systems by combining linear programming (LP) with an ELM. Vergara [15] applied two machine learning techniques to predict active electrical power in buildings by comparing ELM against multilayer perceptron methods. Yeom [16] proposed a new design method based on an ELM with automatic knowledge representation from numerical datasets for short-term electricity-load forecasting. ELM has been applied to water resource management, including discharge forecasting, future projection-based climate change scenarios using an online sequential ELM (OS-ELM), and algal bloom prediction using an ELM on reservoirs. Yadav [17] applied OS-ELM as a new technique capable of updating the model equation based on new data entry without much increase in computational cost; this was used in flood forecasting on the Neckar River, Germany. Yin [18] projected future variability of reference evapotranspiration using an ELM and support vector regression in a mountainous inland watershed in north-west China. Wang [19] proposed a hybrid mechanism modeling method, which synthesized the advantages of an ecological dynamic model and a data-driven model. To obtain an appropriate model, a function model library with key impact factors (IFs) in algal bloom formation was first established, and then Tabu search and a genetic algorithm were applied for model structure optimization and parameter calibration, respectively. Wang [20] constructed a mechanism-based model according to algal bloom nonlinear temporal dynamics to reflect nonlinear dynamic changes in the algal bloom formation mechanism. However, studies have not focused on predicting algal blooms using machine learning with water quality and climate data in regulated rivers. Wang [21] proposed an integrated variable fuzzy evaluation model, which has the precision of the algorithm and operability for the assessment of river water quality based on case studies of reservoir and river water. Olyaie [22] compared various artificial intelligence methods, particularly ANNs, adaptive neuro-fuzzy inference system, and coupled wavelet and neural network for estimating the suspended sediment load in a river system. Fotovatikhah [23] applied flood management systems, which are the most promising approaches with respect to accuracy and error rate for flood debris forecasting and management. In this study, we attempt to apply ELM-based models to predict chlorophyll-a concentration as an indicator of algal blooms, which was monitored on a weekly basis in four weirs in the Nakdong River, South Korea. Despite a growing awareness of the problems associated with algal blooms in rivers, and particularly in regulated rivers, the drivers of bloom formation and abundance in rivers are not well understood [24]. The Nakdong River is a regulated river that includes many artificial weirs. There has been more social interest in this topic after the construction of weirs in the Nakdong River, and algae blooms have occurred more often downstream rather than upstream. It has also been difficult to predict algal blooms using numerical models. Thus, it is important to rapidly and accurately predict algal blooms. It is more difficult to understand and predict algal blooms in regulated rivers that are connected in sequence because hydrodynamics and water quality are more diverse than in other rivers. ELM models were applied to 4 weirs in the Nakdong River to explore the best input structure and develop a model describing weekly chlorophyll-a concentration while considering parameter minimization, water quality monitoring, and overfitting. Upstream chlorophyll-a concentration data was used in our ELM2 model to improve algal bloom prediction performance. In contrast, the ELM1 model only uses downstream chlorophyll-a concentration data. The ELM1 and ELM2 models showed good performance compared to the conventional neural network with backpropagation (NN-BP) and adaptive neuro-fuzzy inference system (ANFIS).

This paper is organized in the following manner: Section 2 describes the study area and the water quality variables that are relevant to the Nakdong River. Section 3 describes the ELM architecture and learning method. Section 4 presents our simulation results for algal bloom prediction from water quality and weather data. Finally, concluding comments are presented in Section 5.

## 2. Study Area

**Figure 1:**
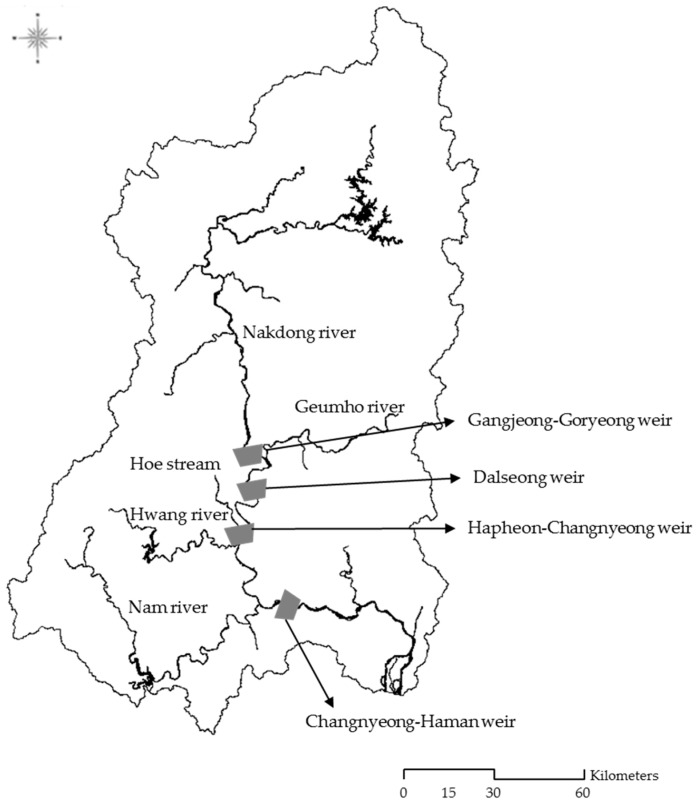
The study area and main streams.

With a length of 525 km, the Nakdong River is the longest river in South Korea, and its watershed area is 23,384 km^2^, which is equivalent to approximately 20% of the country’s area. The Nakdong River has eight weirs which were built in sequence starting in 2012. In particular, four of these weirs (Gangjeong-Goryeong weir, Dalseong weir, Hapcheon-Changnyeong weir, and Changnyeong-Haman weir) in the mid-lower Nakdong River region experience harmful algal blooms every summer, causing many problems for agricultural, residential, and commercial water supplies. Harmful algal blooms refer to toxic, hypoxia-generating cyanobacterial bloom genera controlled by the synergistic effects of nutrients (nitrogen and phosphorus), light, temperature, water residence, and biotic interactions [25]. Since the construction of the weirs, the public and the government have been interested in managing algal blooms. Figure 1 shows the locations of the four weirs on the Nakdong River in South Korea and the watershed area.

All data in this study were obtained from the Korean governmental database system, including the Water Environment Information System and Korea Meteorological Administration System. Monitoring stations were located 500 m upstream from each weir, and water quality data were gathered weekly since weir construction. Chlorophyll-a was used as the primary indicator for algal blooms, and other water quality variables were monitored, including water temperature, pH, dissolved oxygen, electrical conductivity, total nitrogen, nitrate, ammonia nitrogen, total phosphorus, phosphate, biological oxygen demand, chemical oxygen demand, and total organic carbon. In the Gangjeong-Goryeong weir, chlorophyll-a concentration was 19.0 μg/L on average and 106.7 μg/L maximum. In the Dalseong weir, chlorophyll-a concentration was 26.0 μg/L on average and 104.1 μg/L maximum. In the Hapcheon-Changnyeong weir, chlorophyll-a concentration was 23.2 μg/L on average and 100.7 μg/L maximum. In the Changnyeong-Haman weir, which is located downstream, the average chlorophyll-a concentration was the highest at 25.2 μg/L and 123.3 μg/L maximum. Table 1 shows chlorophyll-a, total nitrogen, and total phosphorus statistical values for the 4 weirs from 2013 to 2016. Correlation analysis results showed that correlation coefficient between total nitrogen and chlorophyll-a concentration was 0.263 with a positive correlation, the correlation coefficient between total phosphorous and chlorophyll-a was −0.013 with a negative correlation, the correlation coefficient between N/P ratio and chlorophyll-a was 0.092 with a positive correlation in Gangjeong-Goryoung weir. In the Changnyoung-Haman weir, the correlation coefficient between total nitrogen and chlorophyll-a was −0.036 with a negative correlation, the correlation coefficient between total phosphorous and chlorophyll-a was 0.144 with a positive correlation, the correlation coefficient between N/P ratio and chlorophyll-a was −0.239 with a negative correlation.

**Figure 2:**
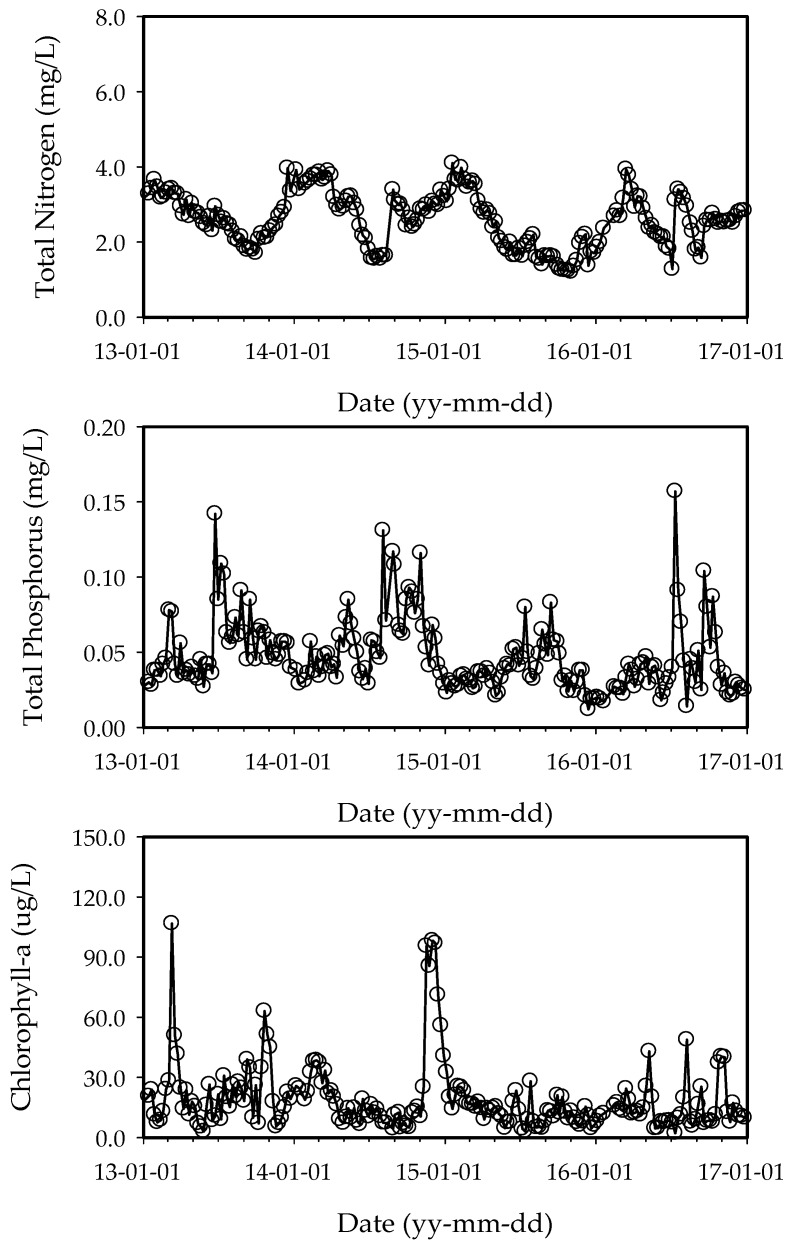
Weekly total nitrogen, total phosphorus, and chlorophyll-a data at the Gangjeong-Goryeong weir from 2013 to 2016 (*n* = 201).

**Figure 3:**
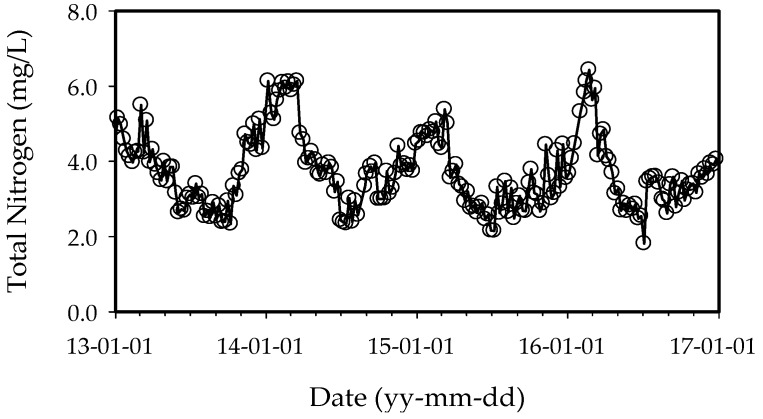

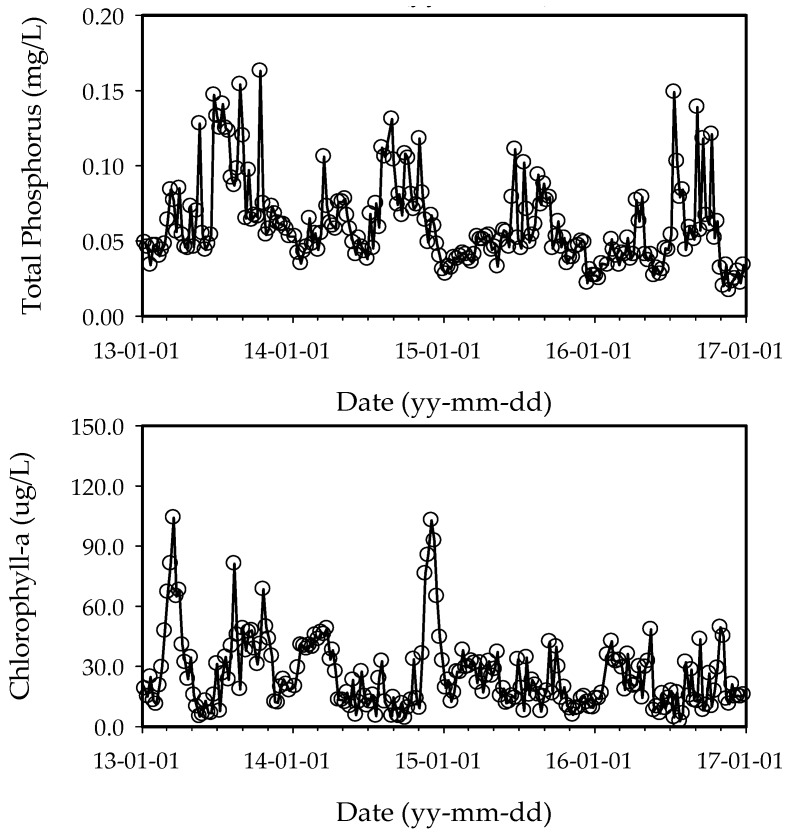
Weekly total nitrogen, total phosphorus, and chlorophyll-a data at Dalseong weir from 2013 to 2016 (*n* = 205).

There are many methods for classifying ecosystems into trophic categories using nutrients and algal biomass. The boundaries placed between these categories by aquatic scientists are similar, but they are not universal. The United States Environmental Protection Agency (US EPA) has suggested that an annual average chlorophyll-a concentration exceeding 10 μg/L indicates a eutrophic state [26]. The 4-year average total phosphorus concentration in the Nakdong River ranged between 19.0 and 26.0 μg/L. Forsberg and Ryding suggested that annual average total nitrogen concentration values ranging from 0.6 to 1.5 mg/L also indicates a eutrophic state [27]. The 4-year average total nitrogen concentration in the Nakdong River ranged from 2.605 to 3.723 mg/L. Also, The Organization for Economic Co-operation and Development (OECD) suggested that an annual average total phosphorus concentration exceeding 0.035 mg/L indicates a eutrophic state [28]. The 4-year average chlorophyll-a concentration in the Nakdong River varied between 0.048 and 0.061 mg/L. Thus, the Nakdong River can be considered to be in a eutrophic state based on all three metrics. Figure 2 and Figure 3 show water quality variations in the Gangjeong-Goryeong and Dalseong weirs from 2013 to 2016.

## 3. Extreme Learning Machine

### 3.1. Architecture and Learning Method for ELM

**Figure 4:**
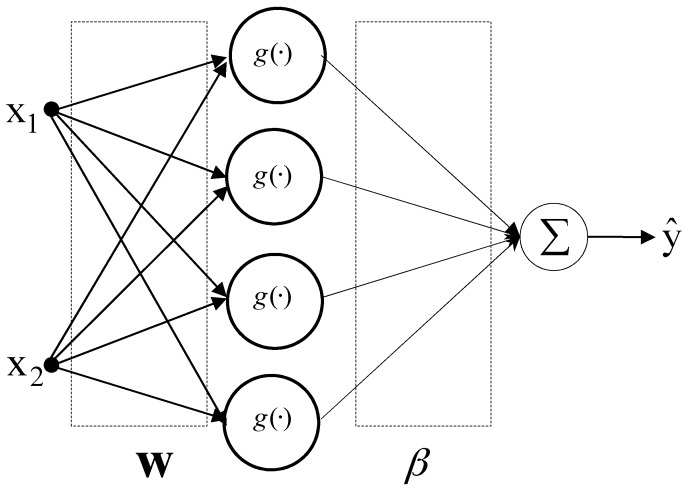
Architecture of the conventional ELM predictor.

ELM was originally proposed as a learning scheme for single hidden layer feedforward neural networks (SLFNs). It was later extended to generalized SLFNs, where the hidden layer need not be neuron-like [29,30]. In the past, gradient-descent-based approaches were used for feedforward neural networks, where all parameters required tuning, which usually requires significant time. ELM has only one hidden layer, and the parameters of this hidden layer need not be tuned, including the input weights and hidden node biases. On the contrary, these hidden node parameters are assigned randomly, which means that they may be independent of the training data. ELM speed can be thousands of times faster than traditional feedforward network learning algorithms with backpropagation while obtaining better generalization performance. The ELM structure has input, hidden, and output layers, as shown in Figure 4.

The ELM model has advantages regarding real-time learning and good prediction capability. The output function from generalized SLFNs is expressed as:

(1) $$f_{L}(x)=\sum_{i=1}^{L}\beta_{i}G_{i}(w_{i},b_{i},x)$$

where **w***_i_* and *b_i_* are the weight and bias between input layer and hidden layer, respectively. The output weights *β* are parameters to be estimated.

The output function in the hidden layer mapping is as follows:

(2) $$H(x)=\left[G_{1}(w_{1},b_{1},x),\cdots ,G_{L}(w_{L},b_{L},x)\right]$$

The output functions of the hidden nodes can be used by various activation functions. The well-known activation functions are sigmoid networks, Radial-Basis Function (RBF) networks, polynomial networks, complex networks, and sine function as follows:

$$\mathrm{Sigmoid}:\;G(w_{i},b_{i},x)=g(w_{i}\cdot x+b_{i})$$

$$\mathrm{RBF}:\;G(w_{i},b_{i},x)=g(b_{i}\left\|x-w_{i}\right\|)$$

$$\mathrm{Sine}:\;G(w_{i},b_{i},x)=\sin(w_{i}\cdot x+b_{i})$$

where conventional random projection is just a specific case of ELM random feature mapping when a linear additive hidden node is used. It not only proves the existence of the networks, but it also provides learning solutions.

In what follows, we shall review the processing procedure of an ELM as a predictor. For a training set, given the activation function and hidden node number, the ELM algorithm can be summarized as three steps: (1) randomly generate the input weights, (2) calculate the hidden layer output matrix, and (3) calculate the output weights matrix. In marked contrast to traditional learning algorithms, ELM requires no iterative adjustment of the network parameters during training. Given a training set $\left\{\left(x_{i},t_{i}\right)|x_{i}\in R^{d},t_{i}\in R^{m},i=1,2,\cdots ,N\right\}$, hidden node output function G(w,b,x), and number of hidden nodes L, the ELM determines the hidden node parameters and output weights using the following steps:

(Step 1) Randomly assign hidden node parameters $(w_{i},b_{i}),i=1,2,\cdots ,N$
(Step 2) Calculate the hidden layer output matrix $H=\left[\begin{array}{c} h(x_{1})\\ \vdots \\ h(x_{N}) \end{array}\right]$
(Step 3) Calculate the output weights *β* using a least squares estimate (LSE):
  (3) $$\beta =H^{*}T$$
  where $H^{*}$ is the Moore-Penrose generalized inverse of matrix H. When $H^{T}H$ is nonsingular, $H^{*}={\left(H^{T}H\right)}^{-1}H^{T}$ is the pseudo-inverse of H. This is a standard LSE problem, and the best solution for *β* is expressed as follows [16]:
  (4) $$\beta ={\left(H^{T}H\right)}^{-1}H^{T}T$$

### 3.2. Model Application

Water quality and weather data in the Nakdong River were collected weekly from January 2013 to December 2016 by the Ministry of Environment, Korea Meteorological Administration. We excluded 2012 and 2017 because the study area had an unstable ecosystem in 2012 during the early period after weir construction. A low water level was maintained intermittently due to social and political issues in 2017. The dataset includes daily air temperature, rainfall, and solar radiation, as well as weekly total nitrogen, total phosphorus, N/P ratio (ratio of total nitrogen to total phosphorus) and chlorophyll-a as inputs to the ELM model. The weekly chlorophyll-a concentration was used as a model output, which is the primary indicator of algal blooms. Parameters like phosphate, nitrate, ammonia nitrogen, which were used for predicting algal blooms, were not applied for model training due to model input minimization and optimization [6,7,8,9]. Table 2 shows input variations, periods, and sources.

**Figure 5:**
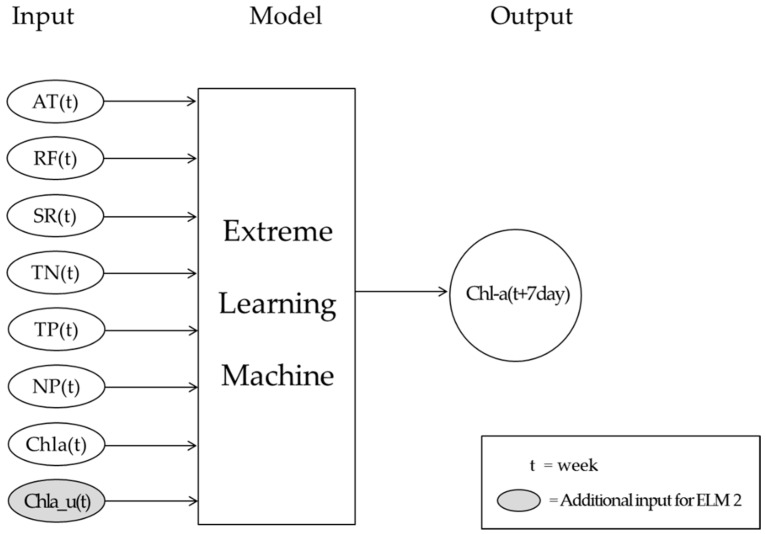
Diagram for ELM model (ELM2). AT: air temperature; RF: rainfall; SR: solar radiation; TN: total nitrogen; TP: total phosphorus; NP: ratio of total nitrogen over total phosphorus; Chla: chlorophyll-a concentration; Chla_u: upstream chlorophyll-a concentration.

The ELM1 model was applied to predict algal blooms using total nitrogen, total phosphorus, N/P ratio, temperature, precipitation, solar radiation, and chlorophyll-a concentration as independent parameters, and the ELM2 model used upstream chlorophyll-a as an independent parameter to improve the predictive power. Figure 5 shows the diagram of the ELM model (ELM2), where t indicates each week, AT is air temperature, and RF is rainfall, SR is solar radiation, TN is total nitrogen, TP is total phosphorus, NP is a ratio of total nitrogen over total phosphorus, Chla is chlorophyll-a concentration, and Chla_u is the upstream chlorophyll-a concentration, respectively. Air temperature data were collected daily and the weekly average values were used, where the 4-year average annual air temperature was 13.4–14.6 °C. Rainfall data were also collected daily and the weekly accumulated values were used, where the 4-year average annual rainfall was 1051–1438 mm/year. Solar radiation data were collected daily and the weekly average values were used for algal bloom prediction, where the 4-year average annual solar radiation was 14.1–14.7 MJ/m^2^. Water quality data with chlorophyll-a concentration were collected weekly and the weekly data were used for algal bloom prediction. Chlorophyll-a concentration was used data from 7 days prior.

50% of the dataset was used for training and the other 50% was used for algal bloom prediction in each weir. The performance of the models in 4 weirs was evaluated using the Pearson correlation coefficient (*R*^2^) and root-mean-square error (RMSE) between the observed and predicted values. These indicators are defined as follows:

(5) $$R^{2}=1-\sum \frac{{\left(Y_{i}-{\hat{Y}}_{i}\right)}^{2}}{{\left(Y_{i}-{\bar{Y}}_{i}\right)}^{2}}$$

(6) $$\mathrm{RMSE}=\sqrt{\frac{1}{n}\overset{n}{\underset{i=1}{\sum }}{\left({\hat{Y}}_{i}-Y_{i}\right)}^{2}}$$

where *n* is the number of data; and $Y_{i}$ are ${\bar{Y}}_{i}$ are observed data and the mean of observed data, respectively, and ${\hat{Y}}_{i}$ is the value predicted from the model.

## 4. Results and Discussion

### 4.1. Experimental Results

**Figure 6:**
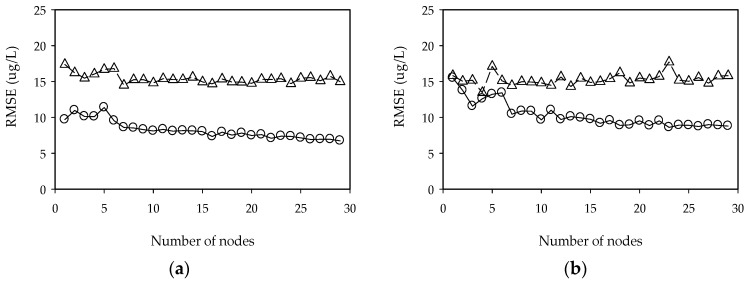
Performance of ELM as a function of the number of hidden nodes. (**a**) Gangjeong-Goryeong weir and (**b**) Dalseong weir.

We applied ELM to chlorophyll-a concentration prediction in four weirs located in the Nakdong River. In the design of ELM, sigmoid networks were adopted as the activation function. We performed the additional experiments for RBF function and sine function. The experimental results revealed that RBF and sine function showed a similar performance in comparison to sigmoid function. ELM models were constructed to determine the optimum number of nodes in the hidden layer. The number of hidden nodes in this study is determined when the performance of test set for model validation reaches a minimum while as the number of hidden nodes increases from 2 to 30. The training and testing performance of the ELM are shown in Figure 6.

**Figure 7:**
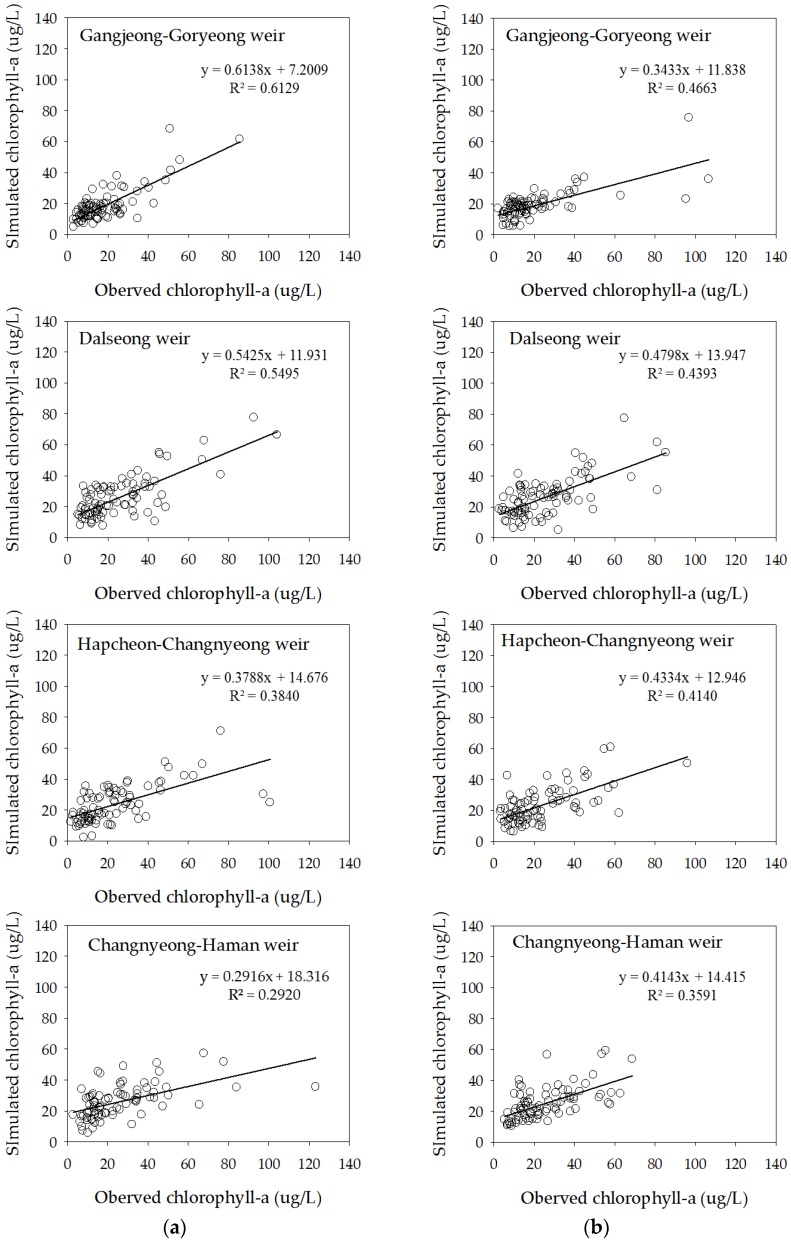
Training and testing results from the ELM1 model for chlorophyll-a prediction. (**a**) Training results and (**b**) testing results.

The performance of the ELM1 models is shown in Table 3 and Figure 7. The prediction results for chlorophyll-a concentration in Gangjeong-Goryeong weir show *R*^2^ = 0.61 for training and 0.47 for testing, and RMSE of 8.6 μg/L for training and 14.5 μg/L for testing. The prediction results in Dalseong weir show *R*^2^ = 0.55 for training and 0.44 for testing, and RMSE of 12.6 for training and 13.5 for testing. The ELM model shows better performance in Gangjeong-Goryeong weir than in Dalseong weir. The prediction results in Hapcheon-Changnyeong weir show *R*^2^ = 0.38 for training and 0.41 for testing, and RMSE of 15.3 for training and 13.1 for testing. The prediction results in Changnyeong-Haman weir show *R*^2^ = 0.29 for training and 0.36 for testing, and RMSE of 16.6 for training and 12.4 for testing. The Akaike information criterion (AIC) was developed for comparing models, based on information theory [31]. AIC applied to Gangjeong-Goryeong weir has a value of 371.2 for training and 452.2 for testing, and 444.6 for training and 455.8 for testing in Dalseong weir. The AIC value in Hapcheon-Changnyeong weir is 461.3 for training and 436.1 for testing data sets, and 469.0 for training and 421.9 for testing data sets in Changnyeong-Haman weir. The predictive power of the ELM model was found to be better in upstream weirs than in downstream weirs. This is because the downstream Nakdong River has more algal blooming factors, such as tributaries, water intakes, and dam discharge, which are difficult to control and manage.

**Figure 8:**
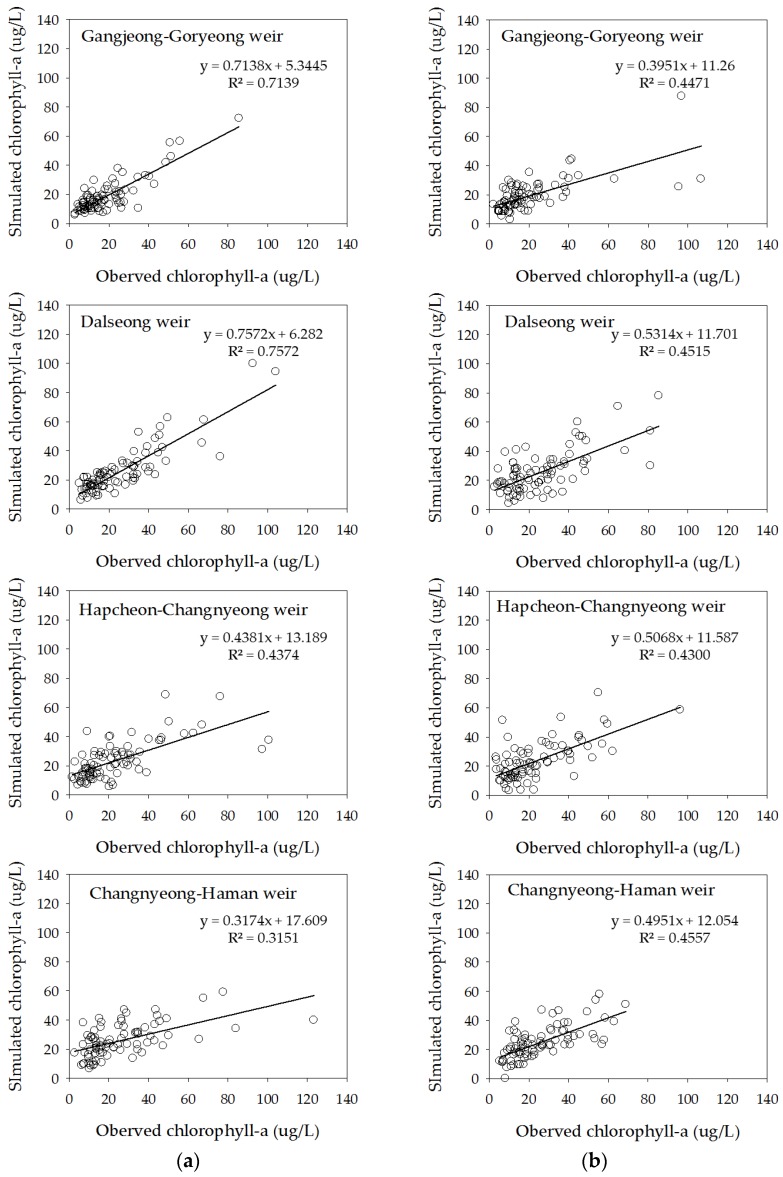
Training and testing results from the ELM2 model for chlorophyll-a prediction. (**a**) Training results and (**b**) testing results.

To improve the accuracy of the chlorophyll-a concentration prediction model, the upstream chlorophyll-a concentration was used as an independent variable in the ELM2 model. The ELM2 (ELM1) model showed better performance with *R*^2^ = 0.71 (0.61) for training and 0.45 (0.47) for testing, RMSE = 6.8 (8.6) for training and 13.8 (14.5) for testing, and AIC = 333.8 (371.2) for training and 452.2 (446.2) for testing in Gangjeong-Goryeong weir. The ELM2 (ELM1) model showed better performance with *R*^2^ = 0.76 (0.55) for training and 0.45 (0.44) for testing, RMSE = 8.9 (12.6) for training and 13.4 (13.5) for testing, and AIC = 388.1 (444.6) for training and 456.9 (455.8) for testing in Dalsone weir. Table 4 and Figure 8 show the results from the ELM2 model in Gangjeong-Goryeong weir and Dalseong weir.

**Figure 9:**
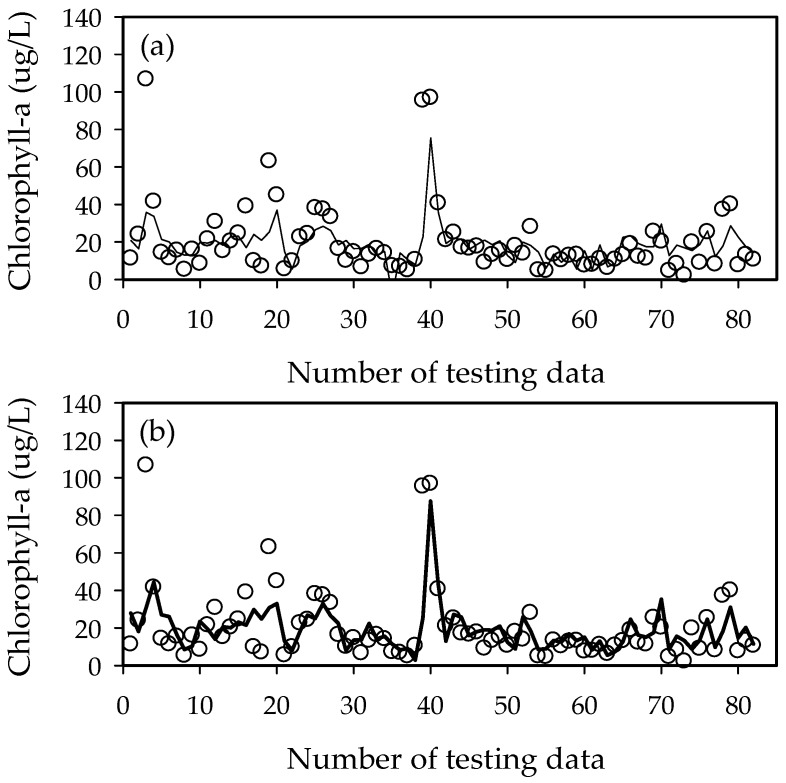
Performance of the ELM1 and ELM2 models in Gangjeong-Goryeong weir. (**a**) ELM1 model and (**b**) ELM2 model.

**Figure 10:**
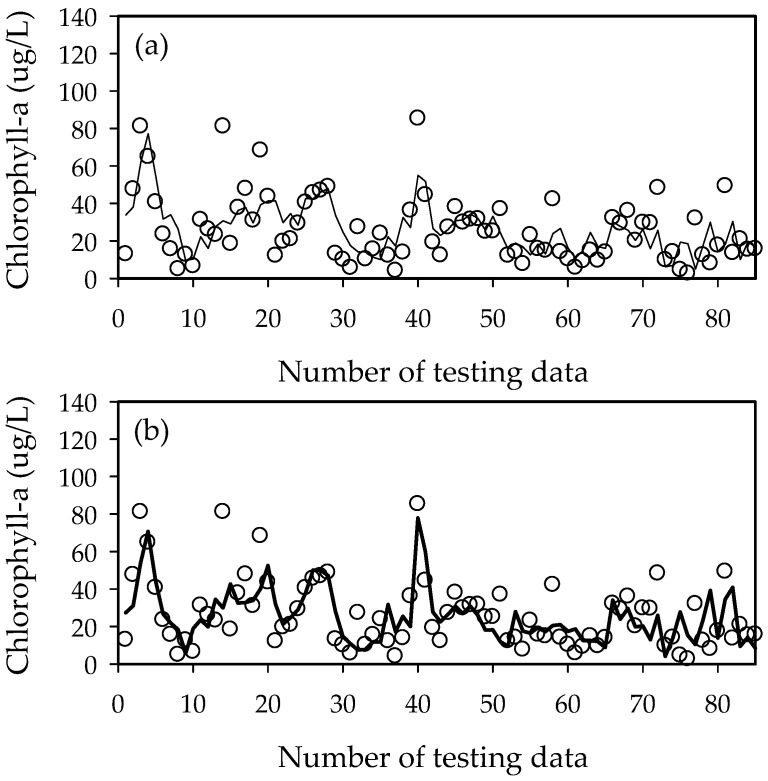
Performance of the ELM1 and ELM2 models in Dalseong weir. (**a**) ELM1 model and (**b**) ELM2 model.

**Figure 11:**
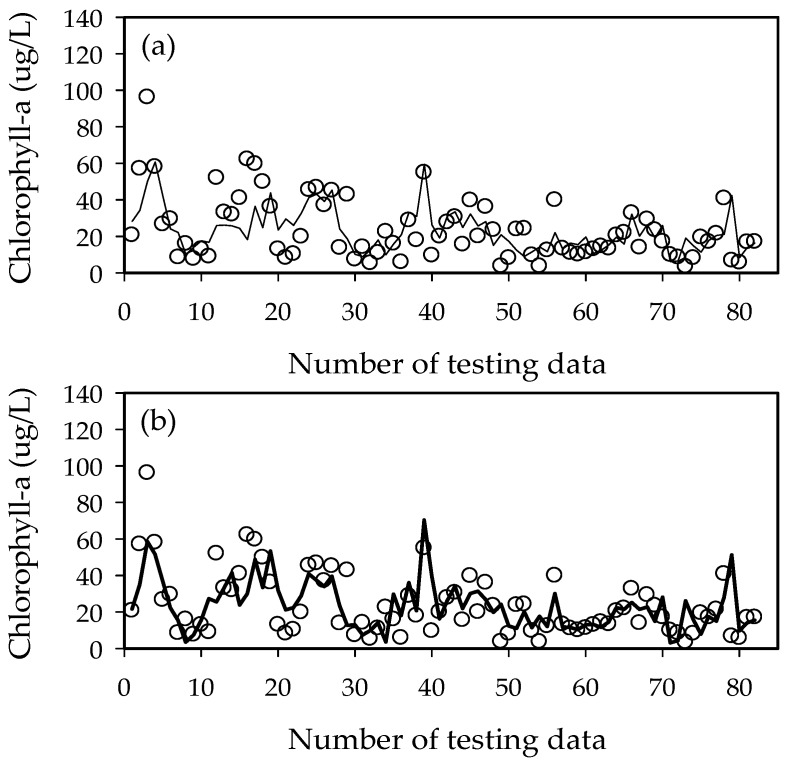
Performance of the ELM1 and ELM2 models in Hapcheon-Changnyeong weir. (**a**) ELM1 model and (**b**) ELM2 model.

**Figure 12:**
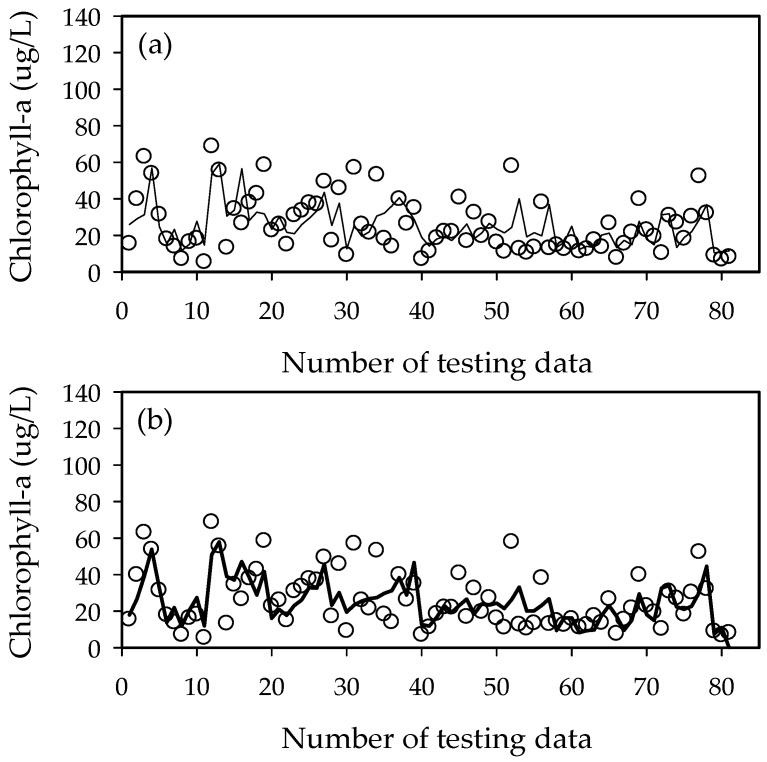
Performance of the ELM1 and ELM2 models in Changnyeong-Haman weir. (**a**) ELM1 model and (**b**) ELM2 model.

**Figure 13:**
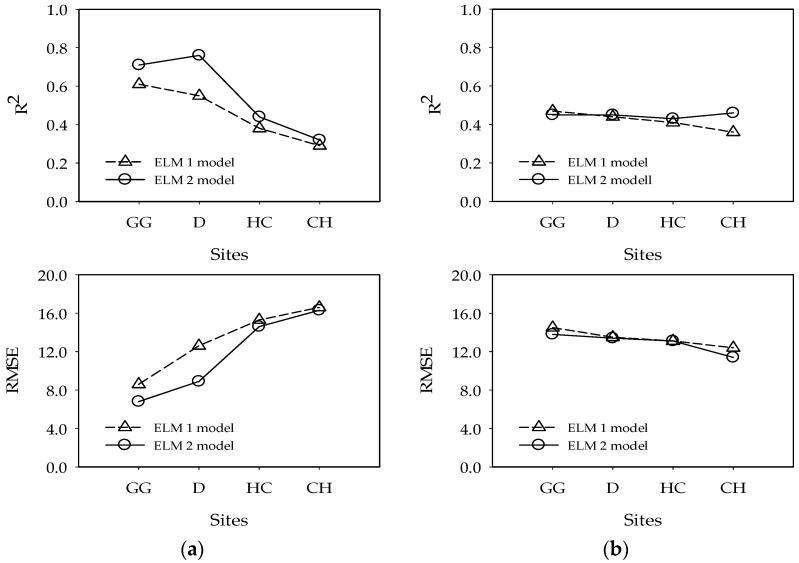
Comparison between the ELM1 and ELM2 model results for chlorophyll-a prediction in all four weirs. (**a**) Training results and (**b**) testing results. GG: Gangjeong-Goryeong weir; D: Dalseong weir; HC: Hapcheon-Changnyeong weir; CH: Changnyeong-Haman weir.

The ELM2 (ELM1) model showed better performance with *R*^2^ = 0.44 (0.38) for training and 0.43 (0.41) for testing, RMSE of 14.6 (15.3) for training and 13.1 (13.1) for testing, and AIC of 455.8 (461.3) for training and 437.5 (436.1) for testing in Hapcheon-Changnyeong weir. The ELM2 (ELM1) model showed better performance with *R*^2^ = 0.32 (0.29) for training and 0.46 (0.36) for testing, RMSE = 16.3 (16.6) for training and 11.4 (12.4) for testing, and AIC = 468.3 (469.0) for training and 410.5 (421.9) for testing in Changnyeong-Haman weir. The ELM2 results from both downstream weirs were similar to the ELM1 model (Figure 9, Figure 10, Figure 11, Figure 12 and Figure 13). This is because the downstream Nakdong River has more algal blooming factors such as tributaries, water intakes, and dam discharge, which are difficult to control and manage. Thus, these algal blooming factors need to be applied to the ELM2 model for more accurate prediction. On the other hand, upstream chlorophyll-a concentration can be a good indicator to predict algal blooms in upstream weirs. Moreover, we compared with the well-known conventional neural network with BP (Back-Propagation) in Table 5. Here, the learning rate was 0.001 and the number of epochs was 1000. In the case of Gangjeong-Goryeong weir, the RMSE values for training and testing set were 9.27 and 15.73, respectively. We also obtained RMSE values of 11.44 and 14.12 for training and testing data in Dalseong weir, respectively. In Hapcheon-Changnyeong weir, the RMSE values for training and testing are 14.69 and 13.43, respectively. We also obtained RMSE values of 16.68 and 11.35 for training and testing in Changnyeong-Haman weir, respectively. Also, we compared with multiple LR (Linear Regression) in Table 5. In the case of Gangjeong-Goryeong weir, the RMSE values for training and testing set were 11.3 and 17.5, respectively. We also obtained RMSE values of 15.3 and 20.7 for training and testing data in Dalseong weir, respectively. In Hapcheon-Changnyeong weir, the RMSE values for training and testing are 14.7 and 13.9, respectively. We also obtained RMSE values of 16.9 and 14.0 for training and testing in Changnyeong-Haman weir, respectively.

Furthermore, we compared with adaptive neuro-fuzzy inference system (ANFIS) frequently used in conjunction with regression and prediction problems. The effectiveness of ANFIS has been demonstrated in real-world application [32,33,34,35,36,37]. This ANFIS has also known as the most representative neuro-fuzzy model [38]. Here fuzzy c-means (FCM) clustering is used to determine fuzzy if-then rules in the design of ANFIS. This ANFIS-FCM program is available in Fuzzy Toolbox of MATLAB R2018a [38]. As listed in Table 5, the experiments are performed as the number of fuzzy rule (r) increases. The ANFIS with four or more fuzzy rules is excluded due to overfitting problems that the number of parameter exceeds the number of training data. The ANFIS-FCM is performed by one-pass based on LSE without learning. The result clearly showed that the generalization capability of ELM2 outperformed that of ANFIS-FCM.

Water residence time was applied as an independent parameter to improve the model performance, and we compared these results with the ELM2 model. The Gangjeong-Goryeong weir results show an RMSE value of 6.2 for training and 14.4 for testing, and the Dalseong results show an RMSE value of 9.6 for training and 13.3 for testing. The Hapcheon-Changnyeong weir results show an RMSE value of 15.3 for training and 13.0 for testing, and the Changnyeong-Haman weir results show 16.2 for training and 11.7 for testing. The performance results including water residence time were similar to the ELM2 model.

The prediction results for Gangjeong-Goryeong weir show that *R*^2^ improved by 16.4% for training and −4.3% for testing, and RMSE improved by 20.9% for training and 4.8% for testing. The prediction results for Dalseong weir show that *R*^2^ improved by 38.2% for training and 2.3% for testing, and RMSE improved by 29.4% for training and 0.7% for testing. The prediction results for Hapcheon-Changnyeong weir show that *R*^2^ improved by 15.8% for training and 4.9% for testing, and RMSE improved by 4.6% for training and 0.0% for testing. The prediction results for Changnyeong-Haman weir show that *R*^2^ improved by 10.3% for training and 27.8% for testing, and RMSE improved by 1.8% for training and 8.1% for testing. Figure 13 shows a performance comparison between the ELM1 and ELM2 model results in all four weirs.

### 4.2. ELM Performance Discussion

As shown in Figure 9, Figure 10, Figure 11, Figure 12 and Figure 13 and Table 5, it was found from these experimental results that ELM showed good performance and generalization capability in comparison to multiple LR, NN with BP, and ANFIS-FCM. Therefore, the features of ELM can be summarized as follows:

- ELM consists of a simple tuning-free three-step algorithm.
- The learning speed of ELM is extremely fast.
- The hidden node parameters are independent of training data. Although hidden nodes are important, they need not be tuned.
- ELM could generate the hidden node parameters before using the training data.
- ELM can be effectively applied to most real-world problems such as compression, feature learning, clustering, regression and classification.

**Figure 14:**
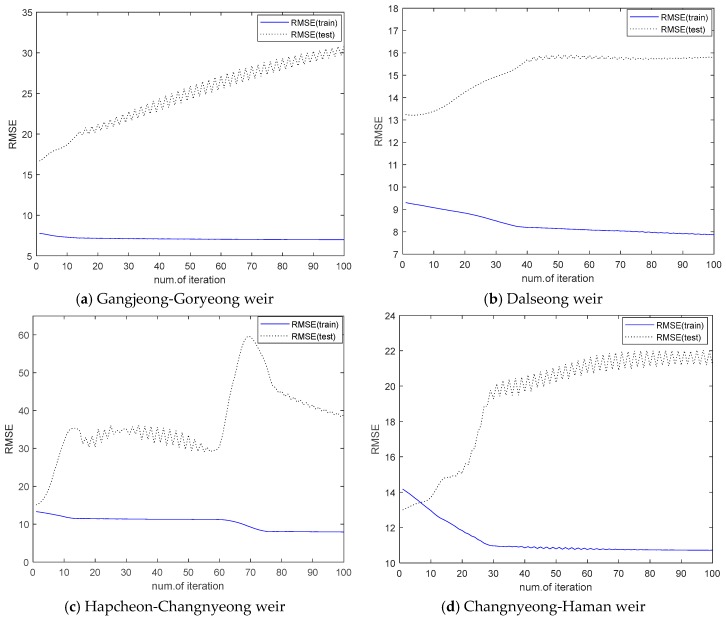
RMSE curves obtained by training of ANFIS-FCM for four weirs (num. of rule = 2). (**a**) Gangjeong-Goryeong weir; (**b**) Dalseong weir; (**c**) Hapcheon-Changnyeong weir; (**d**) Changnyeong-Haman weir.

In general, the superiority of ELM has been demonstrated in comparison with the conventional neural network through real-world applications in the previous literature [8,9,39,40,41]. On the other hand, the ANFIS has been successfully applied to several water quality and algal bloom problems [32,33,34,35,36,37]. With similar experimental methods as previous literatures, we shall perform the experiments on the effects of learning in the design of ANFIS-FCM. Figure 14 shows the error curves for 100 epochs of training in the design of ANFIS-FCM (*r* = 2). The learning method for ANFIS-FCM is performed by forward and backward pass in the hybrid learning procedure based on BP and LSE. As shown in Figure 14, the training error decreased in the case of four weirs, but the test error increased. Usually we use the test error as a true measure of the performance. Thus, the best model is obtained when the test RMSE is minimal. However, although further training decreased the training error, it will degrade the performance of the ANFIS-FCM on unforeseen inputs. That is, the performance after the first epoch is meaningless for this algal bloom data in the case of ANFIS-FCM. For this reason, the ANFIS-FCM was designed by one-pass without learning as the same manner of ELM2 in Table 5. These results lead us to the conclusion that the design of ELM2 for algal bloom prediction is the innovative approach to constructing computationally intelligent model through the performance comparison with the representative models in statistics, neural network, and neuro-fuzzy system, respectively.

## 5. Conclusions

As chlorophyll-a concentration is the primary indicator of algal blooms, an ELM was applied to predict chlorophyll-a concentration in the Nakdong River, Korea. Water quality and weather data were collected on a weekly basis from January 2013 to December 2016 by the Ministry of Environment and Korea Meteorological Administration. Parameters in the dataset include air temperature, rainfall, solar radiation, total nitrogen, total phosphorus, N/P ratio, and chlorophyll-a concentration. 50% of the dataset was used for training, and the other 50% of the dataset was used for testing in each weir. Prediction of chlorophyll-a concentration in Gangjeong-Goryeong and Dalseong weirs, which are located upstream, shows good results for training and testing. Prediction in Hapcheon-Changnyeong and Changnyeong-Haman weirs, which are located downstream, shows worse training and testing results compared to the two upstream weirs. The predictive power of the ELM models was found to be better in the upstream weirs.

To improve the accuracy of the chlorophyll-a concentration prediction model, upstream chlorophyll-a concentration was used as an independent variable in the ELM2 model. Compared to the original ELM1 prediction model, the ELM2 model showed better performance with higher *R*^2^ and lower RMSE values for training and testing datasets in the upstream weirs. The ELM2 model also showed better performance with higher *R*^2^ and lower RMSE values for training and testing datasets in the downstream weirs. However, the results from downstream weirs showed similar performance as the previous ELM1 model. This is because the downstream Nakdong River has more diverse algal blooming factors such as tributaries, water intakes, and dam discharge, which are difficult to control and manage.

ELM-based prediction models for chlorophyll-a concentration in the Nakdong River are proposed in this paper. The purpose of this study is to apply an ELM algorithm for algal bloom prediction in a regulated river with artificial weirs. We included the chlorophyll-a concentration measured from the upstream weir to improve the performance of the algal bloom prediction model. Because we have a small dataset for use in the ELM, we can improve the accuracy of the algal bloom prediction model by examining the water quality in tributaries and accumulating more data in the future. The two ELM models in this study showed superior prediction power, and the upstream chlorophyll-a concentration shows improved prediction accuracy regarding phytoplankton dynamics in a river with sequential weirs. These results showed extreme learning machine can handle more the nonlinearity of algal bloom than linear regression, neural network, and neuro-fuzzy system. Furthermore, these results lead us to the conclusion that the presented ELMs are the effective models for monitoring and managing algal blooms in the regulated river. In future research, we will develop algal blooms prediction models for artificial weirs on the Nakdong and Youngsan rivers in Korea using the Recurrent Neural Network (RNN) and Deep Neural Network (DNN) methods.

## Figures and Tables

**Table 1:** Water quality variables at 4 weirs in the Nakdong River (2013 to 2016).

Variables	Gangjeong-Goryeong Weir	Dalseong Weir	Hapcheon-Changnyeong Weir	Changnyeong-Haman Weir
Chlorophyll-a (μg/L)	19.0 (2.2–106.7)	26.0 (2.7–104.1)	23.2 (1.7–100.7)	25.2 (2.9–123.3)
Total Nitrogen (mg/L)	2.605 (1.201–4.100)	3.723 (1.814–6.433)	3.397 (1.842–6.207)	2.778 (1.249–5.483)
Total Phosphorus (mg/L)	0.048 (0.012–0.157)	0.061 (0.017–0.163)	0.058 (0.016–0.163)	0.054 (0.015–0.174)

Note: Average (Min.–Max.).

**Table 2:** The variables for chlorophyll-a prediction model.

Items	Variables	Source
Weather	Air temperature, Rainfall, Solar radiation	Korea Meteorological Administration (http://kma.go.kr)
Water quality	Total Nitrogen, Total Phosphorus, N/P ratio, chlorophyll-a	Ministry of Environment, National Institute of Environmental Research (http://water.nier.go.kr)

**Table 3:** Statistical analysis of ELM1 model results at four artificial weirs.

ELM1 Model		Gangjeong-Goryeong Weir	Dalseong Weir	Hapcheon-Changnyeong Weir	Changnyeong-Haman Weir
*R* ^2^	Training	0.61	0.55	0.38	0.29
	Testing	0.47	0.44	0.41	0.36
RMSE	Training	8.6	12.6	15.3	16.6
	Testing	14.5	13.5	13.1	12.4
AIC	Training	371.2	444.6	461.3	469.0
	Testing	452.2	455.8	436.1	421.9

**Table 4:** Statistical analysis of ELM2 model at four artificial weirs.

ELM 2 Model		Gangjeong-Goryeong Weir	Dalseong Weir	Hapcheon-Changnyeong Weir	Changnyeong-Haman Weir
*R* ^2^	Training	0.71	0.76	0.44	0.32
	Testing	0.45	0.45	0.43	0.46
RMSE	Training	6.8	8.9	14.6	16.3
	Testing	13.8	13.4	13.1	11.4
AIC	Training	333.8	388.1	455.8	468.3
	Testing	446.2	456.9	437.5	410.5

**Table 5:** RMSE results of other methods comparing ELM2 at four artificial weirs.

Model	RMSE	Gangjeong-Goryeong Weir	Dalseong Weir	Hapcheon-Changnyeong Weir	Changnyeong-Haman Weir
ELM2	Training	6.8	8.9	14.6	16.3
	Testing	13.8	13.4	13.1	11.4
Multiple LR	Training	11.3	15.3	14.7	16.9
	Testing	17.5	20.7	13.9	14.0
NN with BP	Training	9.3	11.4	14.7	16.7
	Testing	15.7	14.1	13.4	11.4
ANFIS-FCM (*r* = 2)	Training	7.8	9.3	13.3	14.2
	Testing	16.7	13.2	15.1	13.0
ANFIS-FCM (*r* = 3)	Training	6.7	8.9	12.9	12.2
	Testing	29.9	16.8	15.2	14.6
